# Quantification of the dark fungal taxon Cryptomycota using qPCR

**DOI:** 10.1111/1758-2229.13257

**Published:** 2024-04-14

**Authors:** Katrin Stüer‐Patowsky, Osu Lilje, Christian Wurzbacher

**Affiliations:** ^1^ Chair of Urban Water Systems Engineering Technical University of Munich Garching Germany; ^2^ School of Life and Environmental Sciences The University of Sydney Camperdown New South Wales Australia

## Abstract

Fungi are present in a wide variety of natural environments, and in the last years, various studies have shown that they are quite abundant in aquatic ecosystems. In addition, a whole new highly diverse phylum, the Cryptomycota, was discovered. Nevertheless, research on aquatic fungi and a detailed evaluation of their functions and distribution are still sparse. One of the main reasons is a limitation in reliable identification and quantification methods. To bridge part of the research gap, this study aims to implement a quantitative PCR method to detect and quantify the newly discovered phylum. We developed and validated a Cryptomycota‐specific qPCR primer pair targeting the 5.8S region that detects the majority of Cryptomycota, but Microsporidia. The resulting amplicon is 102 bp long. We used different environmental samples to evaluate the primer pair, various fungal sequences as negative control and positive control sequences. Obtained amplicons were sequenced using Illumina, and the obtained ASVs were all classified as Cryptomycota. The qPCR method works reliably and specifically for the quantification of Cryptomycota in environmental samples.

## Graphical abstract

In recent years, studies have shown that fungi, including the dark fungal taxon Cryptomycota, are abundant but poorly studied in aquatic ecosystems. We developed and validated a Cryptomycota‐specific qPCR primer pair targeting the 5.8S region that detects most Cryptomycota but not Microsporidia in environmental samples.

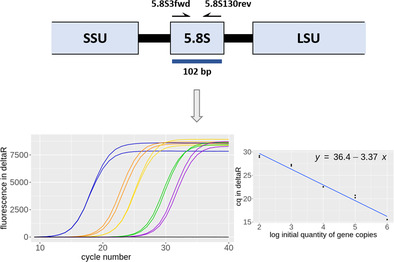

## INTRODUCTION

Studies on aquatic fungi show methodological limitations hampering microbiological and genetic analysis (Frenken et al., 2017; Hanrahan‐Tan et al., 2023). This field of research gained momentum just in the last few years. Therefore, standardized and reliable workflows and sufficient reference data need to be established to close the knowledge gaps comprehensively (Grossart & Rojas‐Jimenez, 2016). Furthermore, cultivation‐based investigation often fails, as substrates or hosts are yet to be discovered. Typical trophic roles of aquatic fungi involve the degradation of allochthonous plant material, like leaf litter, and the parasite‐mediated mineralization of microscopic organisms (Wurzbacher et al., 2010). This biotrophic or saprotrophic lifestyle on organic particles results in a nondescript cell morphology, including zoospores, round‐shaped sporangia, and cysts (Jobard et al., 2010), increasing the possibility of misidentification solely based on morphological characteristics (Lefèvre et al., 2007). DNA barcodes are a more reliable method of detection and diversity analysis. However, the gaps in existing reference databases regarding taxonomic coverage and marker coverage (Wurzbacher et al., 2018) hampers the interpretation of achieved results.

Recent studies showed that fungi are abundant in various aquatic habitats, even revealing a whole new aquatic phylum, the Cryptomycota (Jones, Forn, et al., 2011; Jones, Richards, et al., 2011), or Rozellomycota (Corsaro et al., 2014) (Lara et al., 2010). This phylum is known to be parasitic on fungal or fungus‐like hosts such as Chytridio and Oomycota. (Gleason et al., 2017) but are putative parasites of other hosts. Being closely related to Microsporidia (Corsaro et al., 2014) and Aphelidea, the phyla share ecological and structural features (Wijayawardene, 2020). Microsporidia and Cryptomycota are even proposed to join in the phylum Rozellomycota (Tedersoo et al., 2018). However, although Cryptomycota are primarily parasites of fungi, Aphelides target mainly algae, while Microsporidia infect Metazoa and some protists (Park & Poulin, 2021; Wijayawardene, 2020).

Fungi are known to play a significant role in carbon degradation in the terrestrial environment, while their role in aquatic ecosystems is largely enigmatic. Especially in engineered biological systems like wastewater treatment plants (WWTPs), it is crucial to understand the whole microbial community to enable an optimization of the designed processes. Consequently, including the fungal kingdom in microbial concepts is highly relevant. The newly discovered phylum Cryptomycota has been shown to be the dominant eukaryotic organism in many wastewater systems, anaerobic environments (Chouari et al., 2017; Hirakata et al., 2017), or anaerobic or aerobic trickling filter‐like systems (Miyaoka et al., 2017).

These earlier studies on Cryptomycota relied on molecular cloning or amplicon‐based metabarcoding surveys, which produce relative abundances that are often skewed in their distribution. Relative abundance is based on endpoint amplicons and is therefore only semi‐quantitative in terms of relative abundance and only qualitative with respect to the original sample. A widely used method to enable the quantification of DNA molecules in a sample is qPCR. The execution of qPCR can enable the detection and quantification of reference genes that can be more or less organism‐specific depending on chosen primers. In previous studies, specific primer sets to survey the biodiversity of Cryptomycota were already developed (Lazarus & James, 2015). However, the amplicons produced by those are too long to enable an efficient qPCR quantification. Furthermore, the primers of the study lead to the amplification the zoosporic fungal phylum Chytridiomycota (Quandt et al., 2023). Therefore, there is currently no tool to reliably and specifically quantify Cryptomycota in the environment.

Since Cryptomycota are at the very base of fungal evolution (Grossart et al., 2016), they are expected to show distinct differences in the genetic code compared to other organisms. For most studies, rDNA regions, such as the internal transcribed spacer (ITS), large subunit (LSU), and small subunit (SSU), are used to detect and enable taxonomic studies of fungi (Demirel, 2016). The biggest dataset available for searching suitable regions to target for Cryptomycota specificity contains SSU sequences (Quandt et al., 2023). There are, however, potential sequences covering the LSU and the 5.8S region of the rDNA that need to be considered (Yilmaz et al., 2014). While single‐copy genes would be the preferred choice for a quantitative assay, the currently available genomes are too limited for comparative analysis.

In this study, we aim to implement a quantitative PCR method for Cryptomycota. Regarding Cryptomycota, a wide range of environmental samples, with a particular focus on aquatic habitats, are of interest. Therefore, we will require a specific and reproducible quantification method. Developing and validating specific primer pairs is a crucial step in establishing a reliable qPCR method (Albertsen et al., 2015). We tested several available and newly designed primer options for the ribosomal genes for their robustness and their ability to work with environmental samples and their specificity for Cryptomycota versus relevant outgroups. We confirmed our assay with amplicon sequencing and environmental samples from a wastewater reactor system.

## EXPERIMENTAL PROCEDURES

### Primer design and evaluation

For the primer design and test of existing ones, the ARB software (arb‐6.0.6) was used (Ludwig et al., 2004). As reference databases of the LSU (SILVA_138.1_LSURef_NR99_30_06_20_opt.arb), containing 95, 286 sequences with 236 being Cryptomycota, and SSU (SILVA_138.1_SSURef_NR99_12_06_20_opt.arb), containing 510, 508 sequences with 10 being Cryptomycota, were downloaded on https://www.arb-silva.de/download/arb-files/ (Yilmaz et al., 2014).

RFAM database (http://rfam.xfam.org/, accessed January 2016) and UNITE (version 7) served as input for the 5.8S database. UNITE was processed with ITSx (Bengtsson‐Palme et al., 2013) to extract the 5.8S fragment. The fasta file was aligned versus the reference alignment from RFAM, dereplicated, and screened for ambiguities using Mothur (v.1.31.2, Schloss et al., 2009). This resulted in 6525 sequences derived from UNITE. This initial database was amended by sequences from RFAM. 681,344 5.8S RFAM fasta sequences were processed with Mothur (v.1.31.2) to dereplicate (71,810 unique sequences), trim (minimum of 100 nt), align, filter, precluster at 98%, check for chimeras, and to screen and filter for ambiguous nucleotides, resulting in eventually 20,414 quality filtered 5.8S sequences. Duplicate entries were removed (in RFAM), which resulted in a database with 21,510 sequences. These sequences were used to generate an ARB database (Ludwig et al., 2004). ARB was used to further curate the database. 1660 duplicated sequences were flagged, 655 bacterial entries were removed. FastTree 2 (Price et al., 2010) was used to generate a phylogenetic tree based on maximum likelihood. Entries were then classified using UNITE as reference and the consensus classification from RFAM for maximum likelihood supported clades with missing UNITE entries. Contradicting taxonomies were marked. NCBI taxonomy is taken when no other information is available in the adjunct clades. Eventually, the taxonomy is adapted to the UNITE path taxonomy. A field with ‘low confidence’ or ‘species conflict’ was added. Datafields are: name, full_name, ML_tax, acc, source_db, tax_unite, unite_sh, tax_ncbi, tax_new, low_confidence, species_conflict. The curated ARB database is available in the supplementary (S1). Additionally, Rozellomycota (11,814 sequences) and Aphelidiomycota (140 sequences) sequences of a current update of UNITE (Abarenkov et al., 2023) were used to reassure specific targeting of as many Cryptomycota as possible. Furthermore, NCBI (Sayers et al., 2022) was used to find 178 sequences containing Microsporidian 5.8S genes. To design primers, we screened these three ribosomal databases using the probe design tool in ARB for oligonucleotides with 18–25 nt length for the exclusive detection of Cryptomycota lineages. This was followed by an in silico analysis using Geneious (v 2022.1.1). With the ‘test with saved primers’ and ‘extract PCR products’ functions, the two databases were checked for possible PCR products.

### Sample collection and DNA extraction

Environmental samples were taken from extracted sponge samples of down‐flow hanging sponge (DHS) reactors. These reactors operate comparable to a trickling filter reactor using polyurethane sponges as filling material and are used as wastewater treatment model systems in this study. A defined mass (43 ± 3 mg) of the sponges was taken by prepared punch‐outs and used to extract DNA. The sponge samples were cut into small pieces to prepare for nucleic acid extraction. The DNA extraction was executed using the DNeasy Power Soil Pro kit (Qiagen). The RNeasy Power Biofilm kit (Qiagen) was used for RNA extraction. The concentration of the DNA (DeNovix dsDNA Broad Range Kit, Thermo Fisher Scientific Inc.) and RNA (Qubit RNA Broad Range Assay Kit, Invitrogen [Thermo Fisher Scientific Inc.]) was measured. Ultimately, the DNA was stored at −80°C for long‐term storage. We also tested benthic samples from surface waters derived from Premke et al. (2022) or the local river Isar, Germany. DNA extracts from cultures of the fungal genera *Candida*, *Tetracladium*, *Clavariopsis*, and *Dichotomacladium*, as well as an undescribed strain of Chytridiomycota and a bacterium (*Aquabacterium*) were atested as outgroups.

### Gradient PCR

A gradient PCR was performed to determine optimal annealing temperatures for each primer pair. It was performed in 25 μL reactions with 12.5 μL GoTaq Green Master Mix (Promega GmbH), 5 pmol of each primer, and 1 μL template. We ran an initial denaturation of 2 min at 95°C, then 25 cycles at 95°C for 15 s, 54–64°C for 30 s, and 68°C for 2 min. As no template controls (NTC) nuclease‐free water was used. An 8% gel at 100 V with a runtime of 1 h was used to visually examine the amplicons via gel electrophoresis and GelRed (Merck Millipore, Darmstadt).

### Polymerase chain assembly (PCA)

As a calibration standard for the qPCR oligonucleotides of the in silico found, amplicons were ordered. Since the 5.8S amplicon was short enough, just one could be ordered, while the ones for the SSU primer pair needed to be ordered on parts and then assembled using PCA. Positive controls and standards for the qPCR of the SSU‐ and FungiQuant primer set (Liu et al., 2012) were obtained by ordering several oligomers and assembling them using Polymerase Chain Assembly (PCA). The PCA is performed using two PCR steps. At first, a PCR‐mediated assembly of the oligomer fragments is executed in 25 μL reactions with 12.5 μL GoTaq Green Master Mix (Promega GmbH) and 12.5 μL of template consisting of an equimolar mixture of the fragments. We ran an initial denaturation of 2 min at 95°C, then 20 cycles at 95°C for 30 s, 57.5°C for 30 s, and 72°C for 60 s. The second PCR enabled the gene amplification in 25 μL reactions with 12.5 μL GoTaq Green Master Mix (Promega GmbH), 5 pmol of each primer, and 1 μL of template. We ran 30 cycles at 95°C for 30 s, 58°C for 40 s, and 72°C for 60 s, and the results were examined by gel electrophoresis.

### Quantitative real‐time PCR (qPCR)

All primer sets for Cryptomycota (Table 1) were tested in a 16 μL qPCR assay with 8 μL SSOAdvanced Universal Probes Supermix (Bio‐Rad Laboratories, Inc.), 4.3 pmol of each primer, 2.2 pmol of the probe, and 1 μL of template. Additionally, the primer set targeting the 5.8S region was tested without the additional probe in a 20 μL qPCR assay with 10 μL SSOAdvanced Universal SYBR Green Supermix (Bio‐Rad Laboratories, Inc.), 5 pmol of each primer, and 1 μL of template. The initial activation/denaturation took place for 4 min at 95°C, followed by 35 cycles of a two‐step PCR with a denaturation step at 95°C for 10 s and the corresponding annealing temperature (Table 1) for 20 s. The qPCR was followed by a melting curve analysis (65–95°C in 0.5°C steps) to inspect for unspecific amplifications. All reactions were run and analysed in technical duplicates using a CFX96 thermal cycler (Bio‐Rad Laboratories, Inc.).

**TABLE 1:** Designed and researched primers and probes for quantifying Cryptomycota sets.

Name	Sequence (5′ – 3′)	Source	Empirical T_annealing_ in °C	Amplicon size in bp	Target	Fungal sequences in database	Matches	
							Cryptomyocota	Potential of‐target
SSU334‐F	TACCACTTCCAAGGAAGGCA	This study	58	512	SSU	510,508	219/236 (93%)	48,881
SSU388‐P	FAM‐GAGGTAGTGACAGTAAATAAC‐BHQ	This study						26,186
SSU687‐R	AAATCCAAGAATTTCACCTCT	(Tedersoo et al., 2018)						41,624
5.8S3fwd	CTTTTAACAATGGATCTCTAGGCTC	This study	62	102	5.8S rRNA	20,844	44/52 (85%)	1015
5.8S30probe	FAM‐GCTGCGTTCTTCATCGATGC‐BHQ	(White et al., 1990)						16,580
5.8S130rev	GTTCAAAGATTAGATGACTCACAGA	This study						336
FungiQuant‐F	GGAAAACTCACCAGGTCCAG	(Liu et al., 2012)	60	311	SSU	510,508	230/236 (97%)	33,867
FungiQuant‐P	FAM‐TGGTGCATGGCCGTT‐BHQ							64,857
FungiQuant‐R1	GGTCTATCCCCAGCACGA							18,868
FungiQuant‐R2	GCTCTATCCCCAGCACGA							18,676

*Note*: Including the sequences in the database used for checking specific and unspecific matching (a maximum of 2 mismatches per primer allowed).

### Sequencing of amplicons

We finally evaluated the specificity of amplicons obtained with 5.8S3/130 that resulted from environmental sequences by Illumina sequencing. PCR products derived from the DHS reactor and freshwater lakes were sent to the Z.I.E.L. Institute for Food & Health Core Facility Microbiom (Technical University of Munich) for sequencing. Briefly, the amplicons were adapter‐barcoded 5.8S3fwd‐Ad (5′‐*TCGTCGGCAGCGTCAGATGTGTATAAGAGACAG*CTTTTAACAATGGATCTCTAGGCTC‐3′) and 5.8S130rev‐Ad (5′‐*GTCTCGTGGGCTCGGAGATGTGTATAAGAGACAG*GTTCAAAGATTAGATGACTCACAGA‐3′) and sequenced on an Illumina MiSeq system. The resulting sequencing data was processed using R 4.3.0 in RStudio employing the dada2 (v1.21.0) pipeline (S 2) using UNITE (Abarenkov et al., 2023) for taxon assignment. As filtering parameters truncLen = c (80,80) were used. All 188 ASVs were extracted to align with the 5.8S rRNA database in ARB (arb‐6.0.6) using Muscle (v3.8.3) and implemented in the existing tree using RAxMl (v1.1.0) to see where the sequences cluster.

## RESULTS AND DISCUSSION

### In silico primer screening

This study's overall objective was to identify specific primer regions in known Cryptomycota sequences and subsequently develop a reliable qPCR protocol for quantification. A screening of the LSU, SSU, as well as the 5.8 S region, using ARB, for feasible sequences, revealed relatively few available reference sequences for the SILVA‐SSU dataset (236 sequences Cryptomycota, LKM11, and Rozellomycota), LSU dataset (10 sequences), and 5.8 dataset (52 sequences). We used these references to evaluate three potential primer systems (Table 1), with two potential new primer binding sites in the SSU and two sites in the 5.8S region, and the existing FungiQuant System as a qPCR assay for fungi as a reference (Liu et al., 2012). The LSU region did not result in any potential match, which may have resulted from the few available reference sequences for Cryptomycota. All three primer sets covered most Cryptomycota references. The FungiQuant assay served as a reference targeting fungi in general, which was confirmed by covering a broad diversity of fungal lineages. However, the potential new SSU‐based primer‐probe system also showed a low specificity for the Cryptomycota, leaving the 5.8S primer system with only 21 nontarget organisms, as tested with the in‐silico PCR testing in Geneious. The usage of the 5.8S‐probe did not enhance the predicted specificity of the system, therefore it was neglected in the further assessment. Since a high specificity was one of the main goals in looking for feasible primers, we subsequently focused on using the 5.8S primers. The nontarget matches discovered could primarily be assigned to organisms not typically found in waterbodies (S 3) and were also not recovered by the sequencing below. This search was conducted allowing two mismatches per primer. Mismatches found in the target Cryptomycota sequences were mainly in position 20 (T/A) and positions 12 (C/A), 11 (T/A), 10 (C/T), and 7 (A/G) from the 5′‐end of the forward and reverse primer sequence, respectively (S 4).

The usage of the most current UNITE Rozellomycota sequences with 11,814 sequences as references showed a match for 68.8% excluding Microsporidia, as none of the core Microsporidia sequences retrieved from NCBI showed matches with the 5.8S primer pair. Many Micrsosporidia show a specific rRNA operon structure, being prokaryote‐like in size and structure (16S‐like SSU and 5.8S rRNA genes attached to the 23S‐like LSU) (Corsaro et al., 2019; Vossbrinck & Woese, 1986). This atypical 5.8S structure of the Microsporidia has led to a truncated 5.8S and a missing forward primer‐binding site, which is the reason that Microsporidia cannot be detected with this primer pair.

### In vitro evaluation of the primer pairs

**FIGURE 1:**
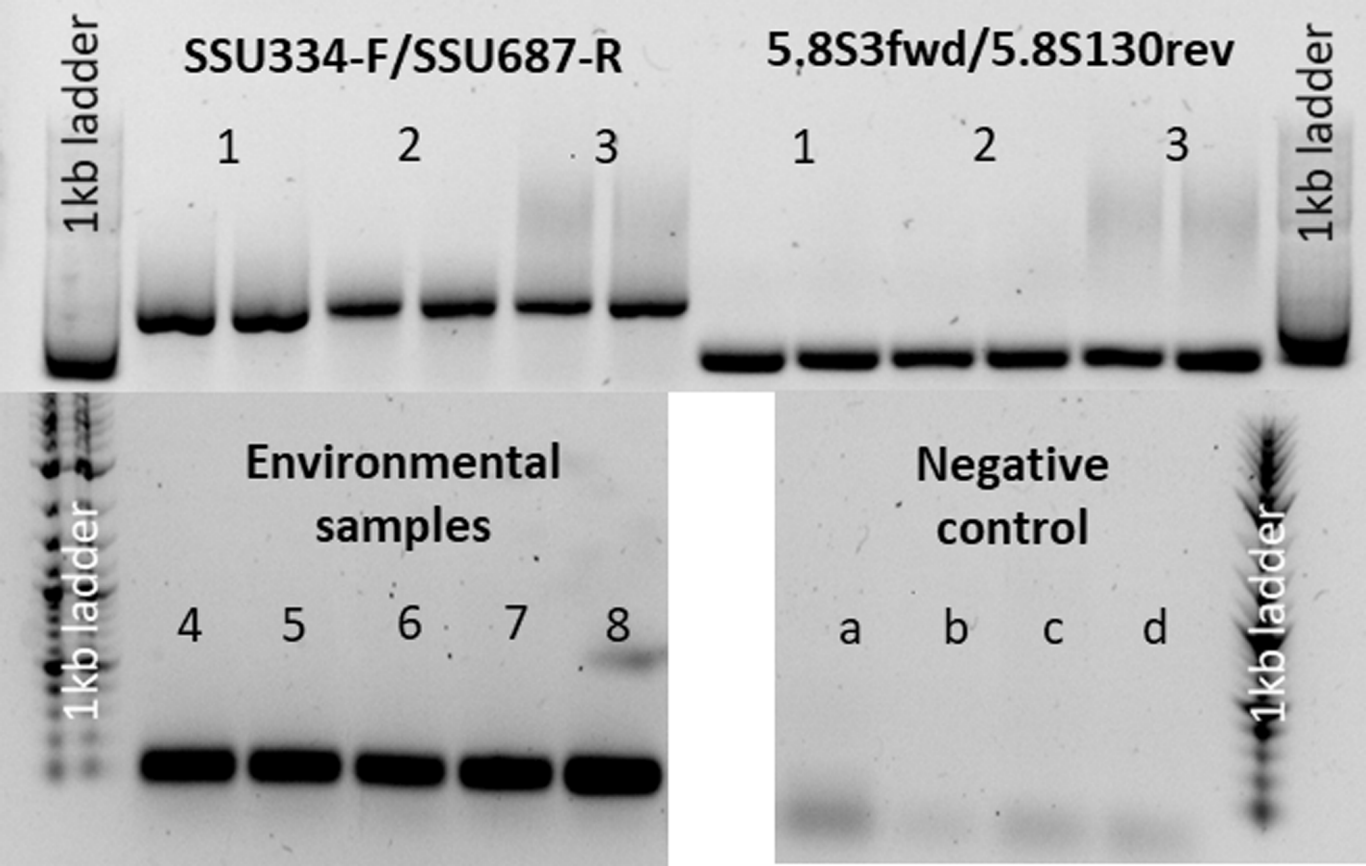
Electrophoresis gel of PCR products. Upper left: Validation of SSU334fwd/SSU687rev primer set. Upper right: Validation of 5.8S3fwd/5.8S130rev primer set. (1) synthetic standard sequence, (2) Isar river water, (3) DHS reactor sponge. Lower left: positive control of 5.8S3fwd/5.8S130rev primer set; (4) Zwenkauer lake, (5) Heinbrauch Königshain (small water body), (6) Fischteich in Lauffen (lake), (7) model system DNA, (8) model system cDNA. Lower right: negative control of 5.8S3fwd/5.8S130rev primer set; (A) *Tetracladium marchalianum*, (B) Chytridiomycota, (C) *Aquabacterium sp*., (D) no template control.

We used a gradient PCR to empirically test the optimum annealing temperature (Table 1) and subsequentially tested its performance with environmental samples. In the study of Miyaoka et al. (2017), the application of a DHS system enabled the enrichment of fungi. The aggregation of biomass and the formation of biofilms provide a positive selection pressure for the organisms of interest. Therefore, samples of pilot‐scale DHS reactors fed with wastewater were used to validate detecting Cryptomycota from environmental samples, along with river water, where Cryptomycota may also occur (Wurzbacher et al., 2015). The synthetic qPCR standards, Isar water, and DHS sponge extracts resulted in valid amplicons for both new primer sets with the expected amplicon length (Figure 1). For the control PCRs that were done with DNA from pure cultures, we could not detect any amplifications for fungal or bacterial DNA for the 5.8S primer pair. In particular, the Chytridiomycota strain, which share the same ecological niche as Cryptomycota as primary aquatic fungi did not show any positive amplification (Figure 1: negative control b). In contrast, the SSU334/687primer pair showed an amplification when DNA from the Chytridiomycota strain was used. Due to these in silico and in vitro observations and the short length (102 bp) of the 5.8S amplicon compared to the SSU amplicon (512 bp), which is favourable for qPCR assays, the 5.8S3/130 primer pair was most suited for its application on environmental samples.

### Evaluation of environmental amplicons

To evaluate the 5.8S primer set even further, we tested two DHS samples and four environmental aquatic samples collected by Premke et al. (2022), who already confirmed the presence of Cryptomycota by metabarcoding in these samples (S 5), for Illumina sequencing of the 5.8S amplicon. All samples showed the expected amplicon length (Figure 1; environmental samples 4 to 8), and we subjected them to Illumina sequencing. In total, 188 ASVs were detected; all of them were assigned to the phylum Rozellomycota (S 6). Further investigation of the phylogenetic clustering revealed that 97.3% of ASVs fell within the Cryptomycota lineages present in our 5.8S database (S 7). Only low abundant ASVs showed spurious clustering within the database (S 8). The apparent identity of a majority and most abundant ASVs with known Cryptomycota leads to the conclusion that the amplicons received by the 5.8S primer‐pair belong indeed to the target organisms. Since Cryptomycota are expected to show approximately a comparable phylogenetic diversity as all so far known fungal genera together (Jones, Forn, et al., 2011; Lazarus & James, 2015), a considerable diversity found in the amplicons was expected.

### Quantitative real‐time PCR

**FIGURE 2:**
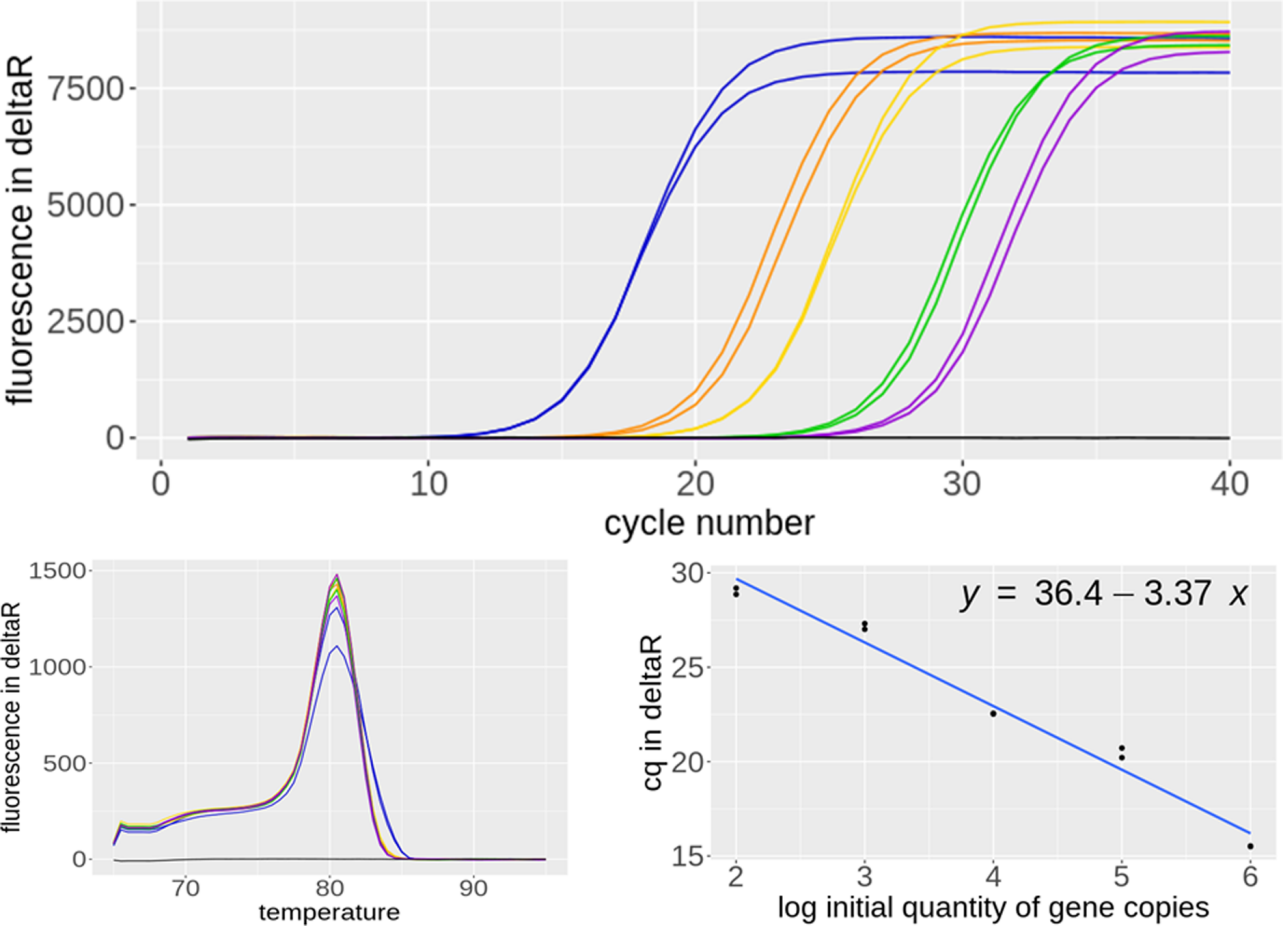
Amplification plots, melting curves, and standard curve from the ordered reference oligomer of the 5.8S primer pair as standard (10^6^ [blue], 10^5^ [orange], 10^4^ [yellow], 10^3^ [green] and 10^2^ [violet] sequence copies) and NTC controls [grey colour].

**FIGURE 3:**
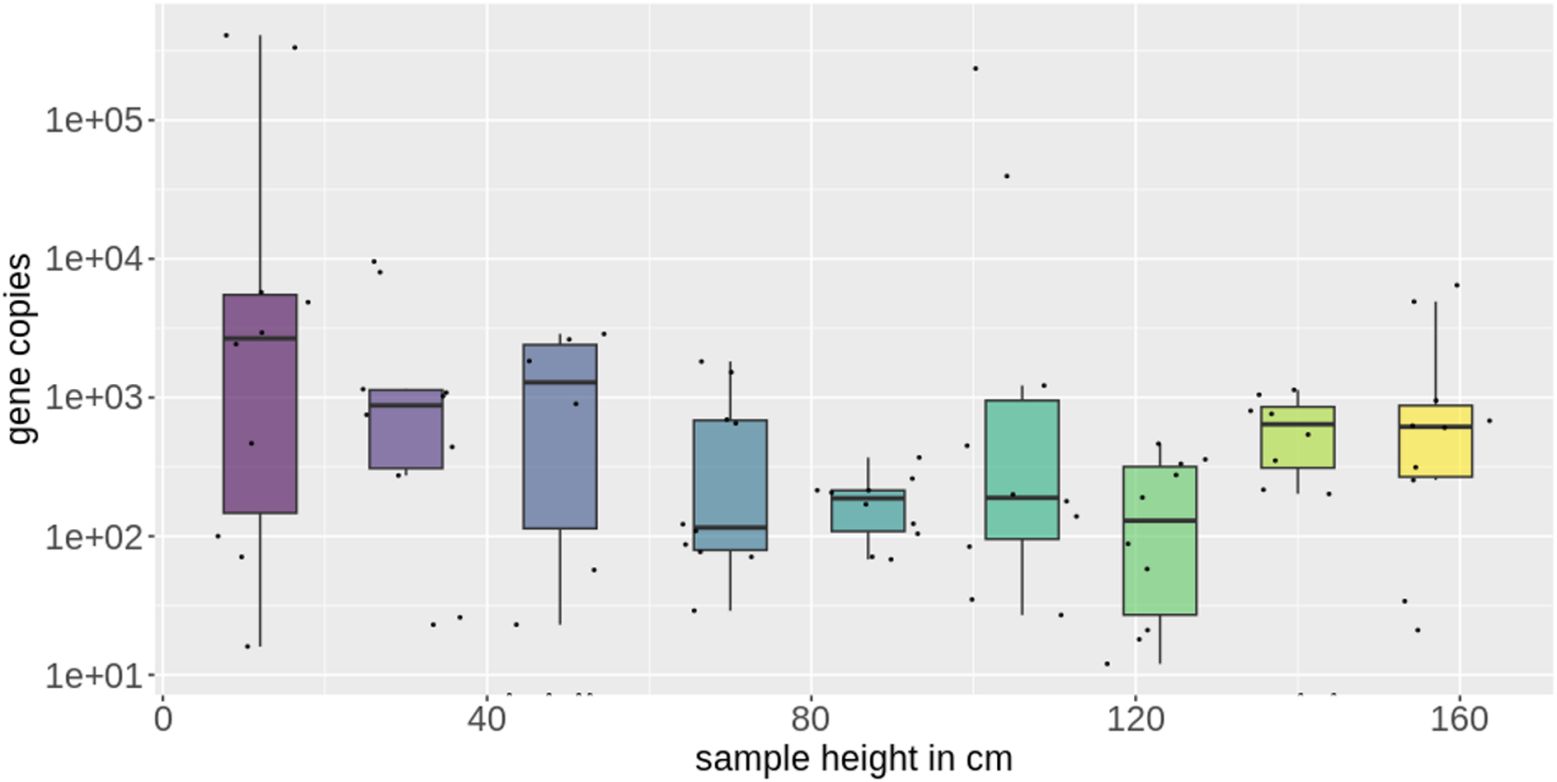
Logarithmic copy number of 5.8S amplicons in DHS reactors as wastewater treatment model systems (*n* = 5) over the reactor height.

For the qPCR, we optimized the cycling times and conditions in order to achieve high efficiency and specificity. This resulted in a final efficiency of 97.96% with an *R* ^2^ value of 0.98 using the standard dilution series (Figure 2), which is in the range of an ideal qPCR performance (Kralik & Ricchi, 2017). We could confirm a robust detection down to 100 copies per reaction. Also, qPCR using the probe assay was tested to verify that the application would be possible (S 9). When applying the developed primer set to our experimental DHS system samples, we could quantify different Cryptomycota abundances within the reactor systems over its height (at 12, 30, 49, 70, 87, 106, 123, 140, and 157 cm) (Figure 3). Overall, a distinct Cryptomycota abundance can be seen in the reactor systems. Further research, including sequencing of the samples is conducted to get insight in the diversity of the Cryptomycota. The results showed that the qPCR method worked on different complex samples and delivered the expected and reproducible outcome.

Overall, the experiments confirmed that the developed 5.8S primer pair leads to reliable and specific detection of Cryptomycota in diverse environmental samples. This is of interest to the following studies for a variety of reasons. Identifying Cryptomycota hotspots and, therefore, relevant sites for further research to gain knowledge of those under‐discovered organisms. The topic of dark fungal taxa is a broad one and has proven to be an evolving field with the need to develop new molecular methods (James et al., 2020; Wijayawardene et al., 2022). Furthermore, the role of fungi in the dynamics of microbial communities in aquatic systems is gaining more interest as a research topic (Ogwugwa et al., 2022). The method developed in this paper provides the possibility to enhance the insight into the systems of those studies by allowing to quantify the Cryptomycota compartment. Since it is already known for the Cryptomycota to be present in a broad spectrum of different aquatic environments (Jones, Forn, et al., 2011; Jones, Richards, et al., 2011), their presence could yield new insights into aquatic food webs and their parasitic role.

## CONCLUSION

To enable the detection and quantification of Cryptomycota using quantitative PCR, a highly specific primer pair targeting the 5.8S region was developed in this study. Neither Aphelidiomycota nor Microsporidia sequences showed in situ amplification. As seen in the qPCR trials, the usage of a probe‐based assay is also possible, although the probe didn't enhance the specificity with the developed primers. Using various complex and diverse environmental samples, it was shown that the developed protocol led to a reliable amplification of the target organisms. Using control samples of common aquatic fungi and amplicon sequencing, the specificity of the primers for Cryptomycota could be confirmed. Aquatic fungi are largely understudied, partly due to methodological limitations. As they are known to be highly abundant in a variety of environmental habitats, it is especially interesting to close our knowledge gaps. Looking into wastewater treatment systems, which environments usually select against fungi, Cryptomycota are present at stable abundances. Other studies already show that getting insight into the fungal community in wastewater treatment could be highly beneficial (Vaksmaa et al., 2023). Fungi are known to produce a wide variety of enzymes that enable the degradation of carbon compounds. It is highly likely that fungi in WWTPs already play an active role in the water treatment system. Understanding the whole microbial community is essential to work with and potentially optimize the processes.

### Next steps

The newly developed method will be a step toward gaining insight into the progressive succession, diversity, and interaction of the fungal community with other microorganisms in the wastewater environment. Using biofilms in down‐flow hanging sponge (DHS) reactors as model systems, future studies will focus on understanding the highly abundant Cryptomycota. In previous studies, the application of DHS systems enabled the enrichment of our organisms of interest.

## AUTHOR CONTRIBUTIONS

**Katrin Stüer‐Patowsky:** Conceptualization (equal); data curation (lead); formal analysis (lead); investigation (lead); methodology (equal); project administration (lead); resources (equal); validation (equal); visualization (lead); writing – original draft (lead); writing – review and editing (equal). **Osu Lilje:** Resources (equal); writing – review and editing (equal). **Christian Wurzbacher:** Conceptualization (equal); funding acquisition (lead); methodology (equal); project administration (supporting); resources (equal); supervision (lead); validation (equal); writing – original draft (supporting); writing – review and editing (equal).

## CONFLICT OF INTEREST STATEMENT

The authors declare no conflicts of interest.

## Supporting information


**Data S1.** Supplementary Information.

## Data Availability

The data for this study have been deposited in the European Nucleotide Archive (ENA) at EMBL‐EBI under accession number PRJEB 69215 (https://www.ebi.ac.uk/ena/browser/view/PRJEB69215).
