# Supplementary material for: Quantification of the dark fungal taxon Cryptomycota using qPCR
Source: Environ Microbiol Rep. 2024 Apr 14;16(2):e13257. doi: 10.1111/1758-2229.13257 (PMC11016352; doi:10.1111/1758-2229.13257)
Supplement: Supplementary file 1 — Data S1. Supplementary Information. [file EMI4-16-e13257-s001.zip › Supplemental_Matierial_S2.docx]

**DADA2 R-script and intermediate output for processing the 5.8S sequencing reads**

#Loading libraries

library(tidyverse) ; packageVersion("tidyverse")

#[1] ‘2.0.0’

library(phyloseq) ; packageVersion("phyloseq")

#[1] ‘1.44.0’

library(vegan) ; packageVersion("vegan")

#[1] ‘2.6.4’

library(DESeq2) ; packageVersion("DESeq2")

#[1] ‘1.40.1’

library(dendextend) ; packageVersion("dendextend")

#[1] ‘1.17.1’

library(viridis) ; packageVersion("viridis")

#[1] ‘0.6.3’

library(dada2); packageVersion("dada2")

#[1] ‘1.21.0’

getwd()

setwd("~/Documents/KSP")

path <- "~/Documents/KSP/fastq"

list.files(path)

# Forward and reverse fastq filenames have format: SAMPLENAME_R1_001.fastq and SAMPLENAME_R2_001.fastq

fnFs <- sort(list.files(path, pattern="_R1_001.fastq", full.names = TRUE))

fnRs <- sort(list.files(path, pattern="_R2_001.fastq", full.names = TRUE))

# Extract sample names, assuming filenames have format: SAMPLENAME_XXX.fastq

sample.names <- sapply(strsplit(basename(fnFs), "_"), `[`, 1)

sample.names

# Inspect read quality profiles

# We start by visualizing the quality profiles of the forward reads:

plotQualityProfile(fnFs[1:2]) # read quality of the first two files in the forward libraries

# Now we visualize the quality profile of the reverse reads:

plotQualityProfile(fnRs[1:2])

# Place filtered files in filtered/ subdirectory

filtFs <- file.path(path, "filtered", paste0(sample.names, "_F_filt.fastq.gz"))

filtRs <- file.path(path, "filtered", paste0(sample.names, "_R_filt.fastq.gz"))

names(filtFs) <- sample.names

names(filtRs) <- sample.names

out <- filterAndTrim(fnFs, filtFs, fnRs, filtRs, truncLen=c(80,80),

maxN=0, maxEE=c(2,2), truncQ=2, rm.phix=TRUE,

compress=TRUE, multithread=TRUE)

head(out)

# reads.in reads.out

#187-DNA87_S187_L001_R1_001.fastq 425601 374673

#188-DNA413_S188_L001_R1_001.fastq 658203 575035

#189-DNA594_S189_L001_R1_001.fastq 601162 526032

#190-T2-10-t_S190_L001_R1_001.fastq 429785 376131

#191-T1-5-4_S191_L001_R1_001.fastq 573609 500606

#193-NC_S193_L001_R1_001.fastq 15 6

#Learn the error rates

errF <- learnErrors(filtFs, multithread=TRUE)

# 118059200 total bases in 1475740 reads from 3 samples will be used for learning the error rates.

errR <- learnErrors(filtRs, multithread=TRUE)

# 118059200 total bases in 1475740 reads from 3 samples will be used for learning the error rates.

plotErrors(errF, nominalQ=TRUE)

# Apply the core sample inference algorithm to the filtered and trimmed sequence data.

dadaFs <- dada(filtFs, err=errF, multithread=TRUE)

#Sample 1 - 374673 reads in 31200 unique sequences.

#Sample 2 - 575035 reads in 39998 unique sequences.

#Sample 3 - 526032 reads in 33739 unique sequences.

#Sample 4 - 376131 reads in 24672 unique sequences.

#Sample 5 - 500606 reads in 27062 unique sequences.

#Sample 6 - 6 reads in 6 unique sequences.

dadaRs <- dada(filtRs, err=errR, multithread=TRUE)

#Sample 1 - 374673 reads in 35523 unique sequences.

#Sample 2 - 575035 reads in 48124 unique sequences.

#Sample 3 - 526032 reads in 37987 unique sequences.

#Sample 4 - 376131 reads in 28190 unique sequences.

#Sample 5 - 500606 reads in 32265 unique sequences.

#Sample 6 - 6 reads in 6 unique sequences.

dadaFs[[1]]

#dada-class: object describing DADA2 denoising results

#159 sequence variants were inferred from 31200 input unique sequences.

#Key parameters: OMEGA_A = 1e-40, OMEGA_C = 1e-40, BAND_SIZE = 16

#Merge paired reads

mergers <- mergePairs(dadaFs, filtFs, dadaRs, filtRs, verbose=TRUE)

head(mergers[[1]])

#abundance forward reverse nmatch nmismatch nindel prefer accept

#Construct sequence table

seqtab <- makeSequenceTable(mergers)

dim(seqtab)

#[1] 6 360

# Inspect distribution of sequence lengths

table(nchar(getSequences(seqtab)))

#Remove chimeras

seqtab.nochim <- removeBimeraDenovo(seqtab, method="consensus", multithread=TRUE, verbose=TRUE)

dim(seqtab.nochim)

#6 188

sum(seqtab.nochim)/sum(seqtab)

# 0.8201801

#Track reads through the pipeline

getN <- function(x) sum(getUniques(x))

track <- cbind(out, sapply(dadaFs, getN), sapply(dadaRs, getN), sapply(mergers, getN), rowSums(seqtab.nochim))

colnames(track) <- c("input", "filtered", "denoisedF", "denoisedR", "merged", "nonchim")

rownames(track) <- sample.names

head(track)

#input filtered denoisedF denoisedR merged nonchim

#187-DNA87 425601 374673 373483 373333 352834 269341

#188-DNA413 658203 575035 573001 572629 529207 438154

#189-DNA594 601162 526032 524670 524306 489416 325512

#190-T2-10-t 429785 376131 375601 375119 314311 293017

#191-T1-5-4 573609 500606 498796 500102 457499 431841

#193-NC 15 6 1 3 0 0

#ETRACTING THE STANDARD GOODS FROM DADA2

# giving our seq headers more manageable names (ASV_1, ASV_2...)

asv_seqs <- colnames(seqtab.nochim)

asv_headers <- vector(dim(seqtab.nochim)[2], mode="character")

for (i in 1:dim(seqtab.nochim)[2]) {asv_headers[i] <- paste(">ASV", i, sep="_")}

# making and writing out a fasta of our final ASV seqs:

asv_fasta <- c(rbind(asv_headers, asv_seqs))

write(asv_fasta, "ASVs.fa")

# count table:

asv_tab <- t(seqtab.nochim)

row.names(asv_tab) <- sub(">", "", asv_headers)

write.table(asv_tab, "ASVs_counts.tsv", sep="\t", quote=F, col.names=NA)

#load data needed

ASVtable <- asv_tab

#SampleInfo <- read.table("SampleInfo16S.tsv")

ASV<-t(ASVtable)

rowSums(ASVtable)

min(rowSums(ASVtable)) #1

#SampleInfo$reads<-rowSums(ASV) # delete low reads

#identify and delete low sample numbers in a data.frame

l=c()

for (i in 1:length(ASVtable[,1])){

if(sum(ASVtable[i,]) < 4000) l=append(l,i)# minimum reads set to 4000

}

#SampleInfo<-SampleInfo[-l,] #delete low read number samples in env matrix

ASV<-ASVtable[-l,] # delete low read number samples in taxa table

rowSums(ASV)

#subsampling

minsum<-min(rowSums(ASV))

minsum

#4099

table.rar <- data.frame(rrarefy(ASV, minsum))

rowSums(table.rar)

#delete empty ASV

table.rar<-table.rar[,(colSums(table.rar) != 0)]

write.table(ASV, "ASVs_counts_4000.tsv", sep="\t", quote=F, col.names=NA)

################################################################

#searching for Cryptomycota

#assign taxonomy (dada2 pipeline)

library(dada2)

taxa <- assignTaxonomy(seqtab.nochim, "~/Documents/sh_general_release_dynamic_25.07.2023.fasta", multithread=TRUE)

taxa.print <- taxa # Removing sequence rownames for display only

rownames(taxa.print) <- NULL

head(taxa.print)

write.csv(taxa, "~/Documents/KSP/taxa_16S_DNA.csv", row.names=TRUE)
