# Supplementary material for: Quantification of the dark fungal taxon Cryptomycota using qPCR
Source: Environ Microbiol Rep. 2024 Apr 14;16(2):e13257. doi: 10.1111/1758-2229.13257 (PMC11016352; doi:10.1111/1758-2229.13257)

*S 3: Nontarget organisms with corresponding habitat potentially amplified by 5.8S primer pair in vitro analyzed using Geneious.*


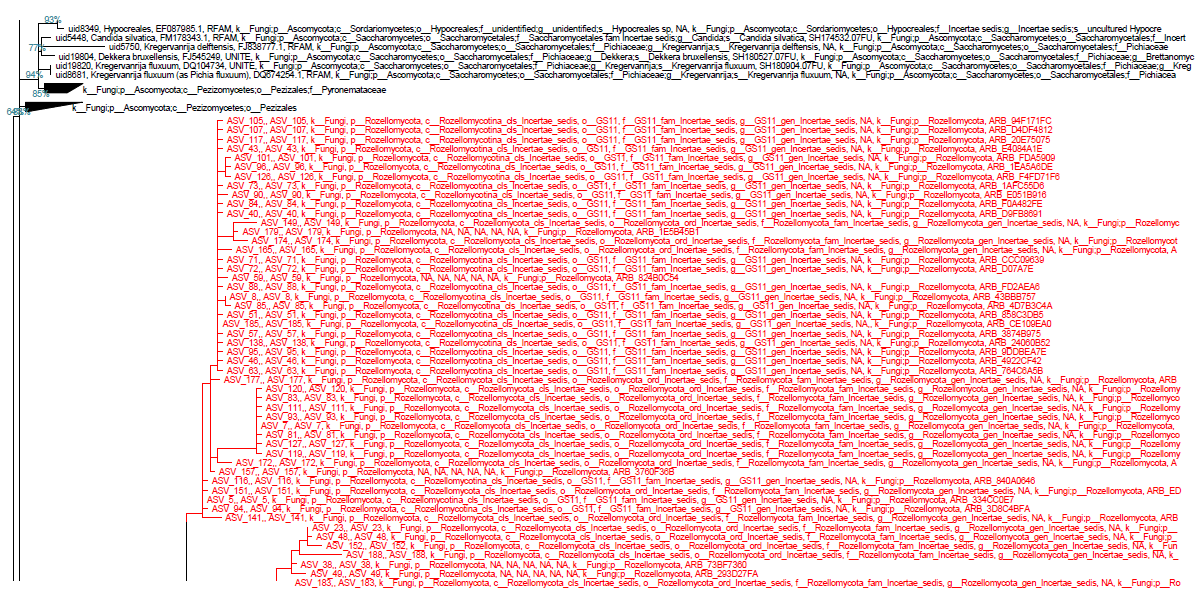

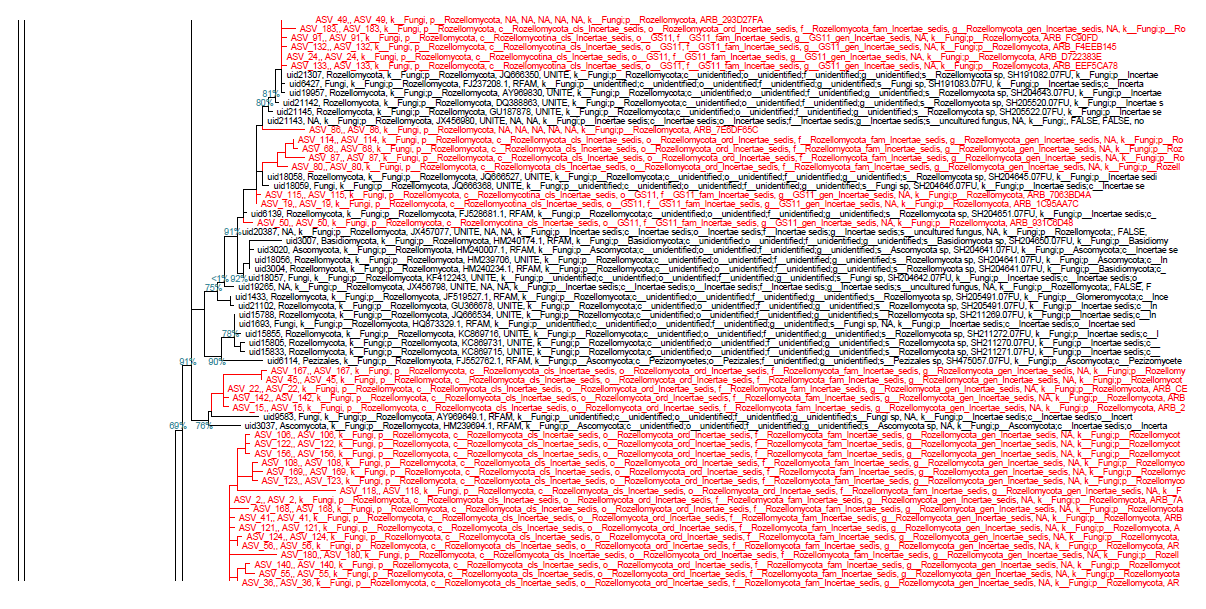

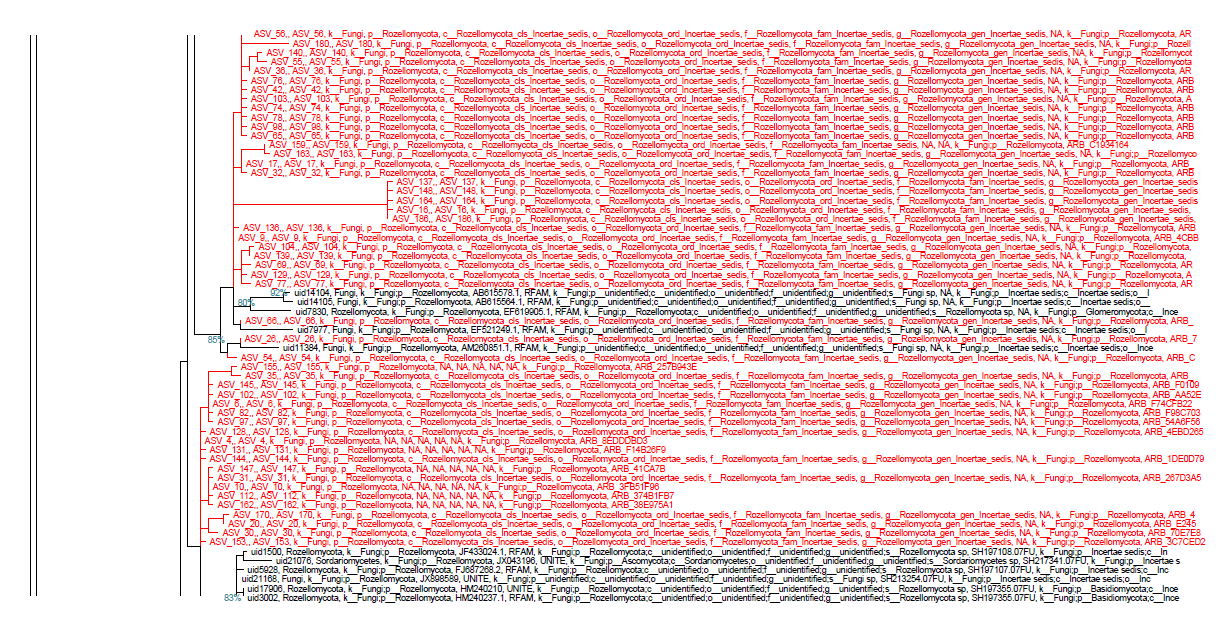

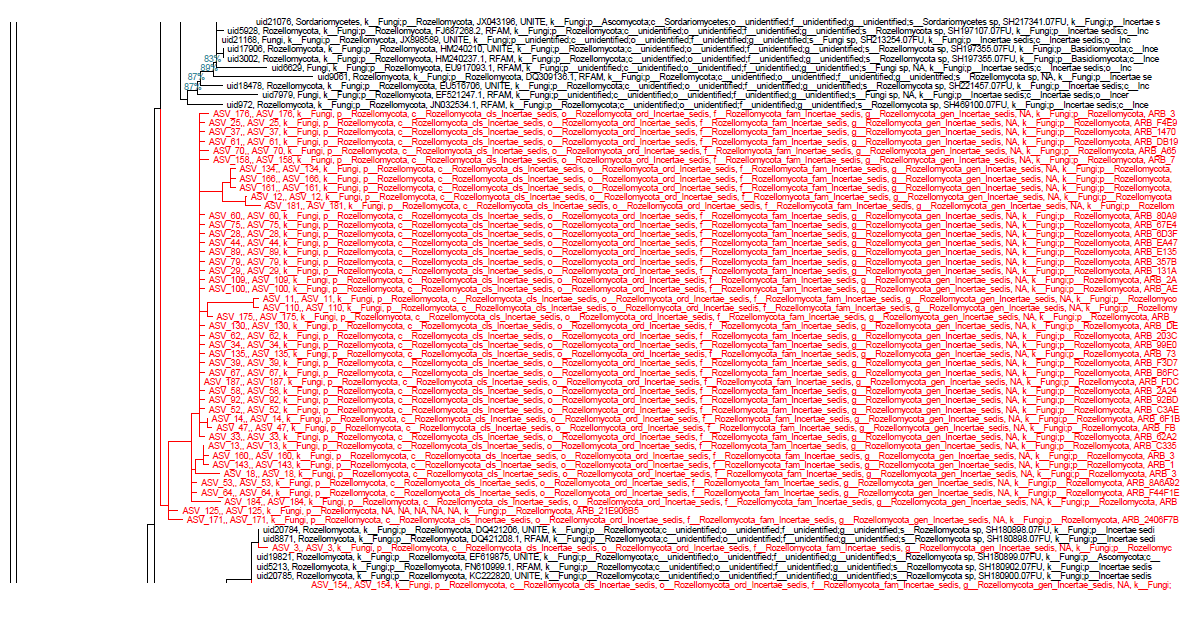

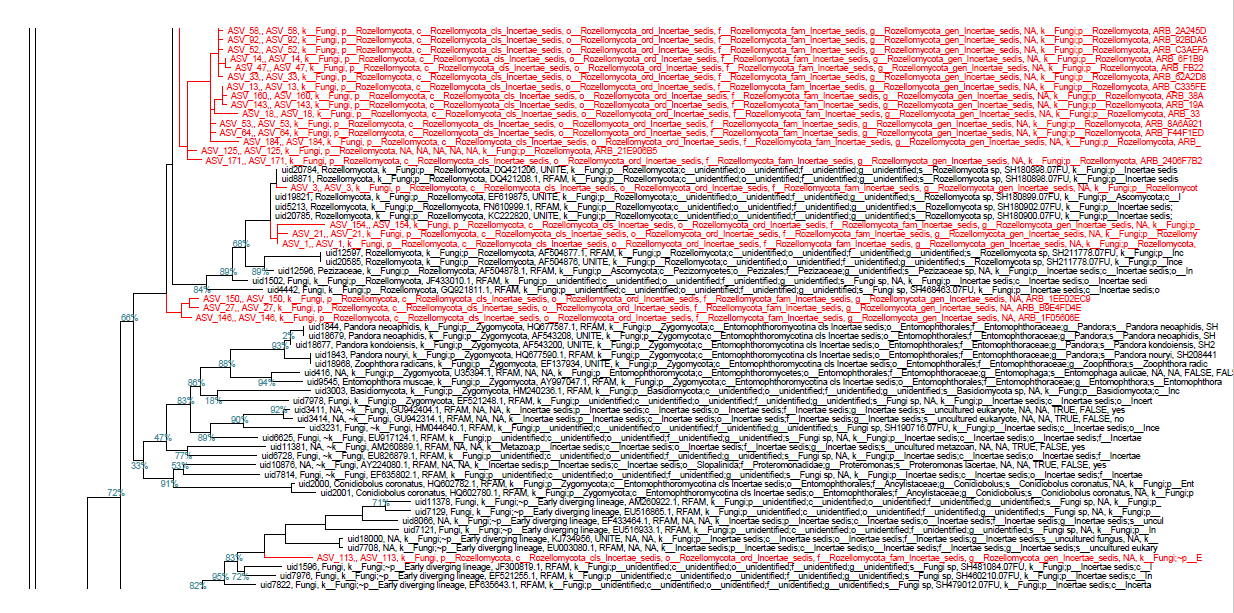


S 7: Extract of the 5.8S database tree shown in arb-6.0.6 with ASV 1 to 188 clustering with Rozellomycota (marked in red).


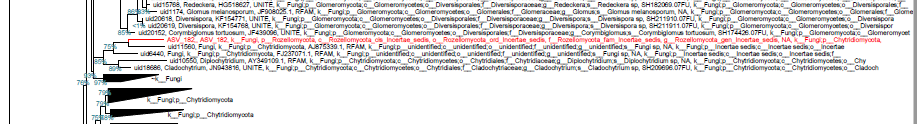


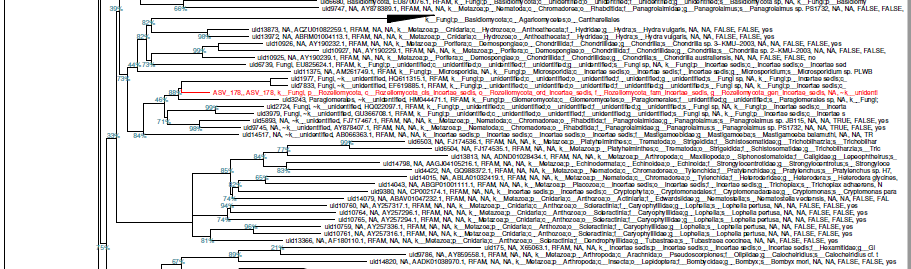


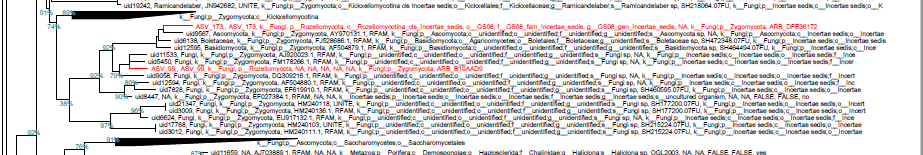


S 8: Extract of the 5.8S database tree shown in arb-6.0.6 with ASV 1 to 188 not clustering with Rozellomycota (marked in red).


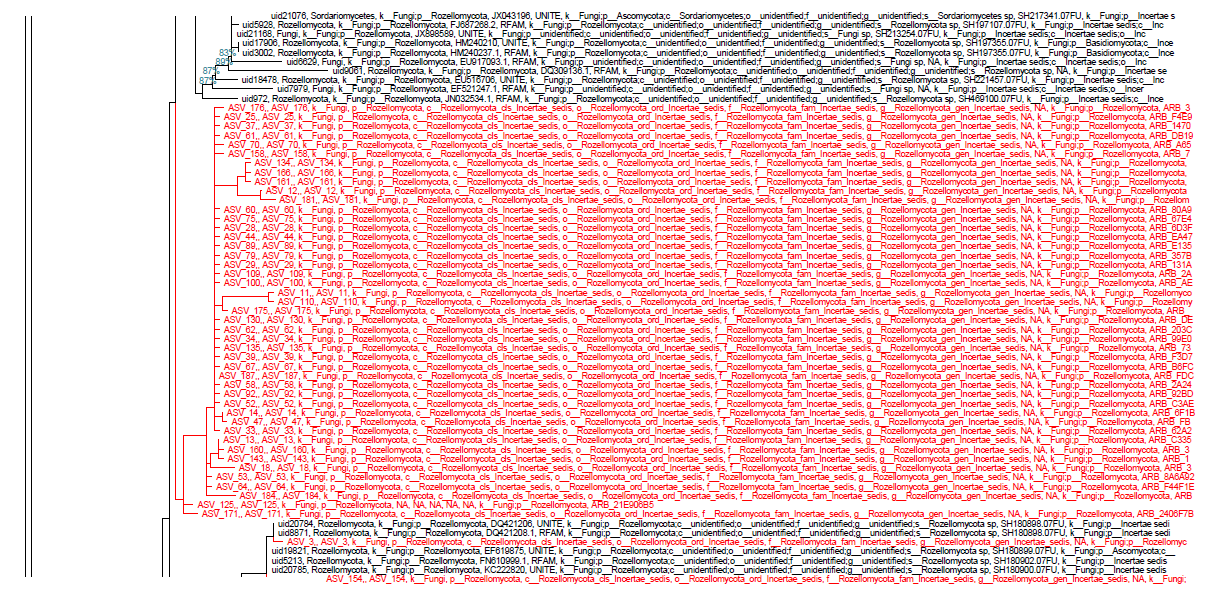


S 9: *Amplification plots, and standard curve using the 5.8S primer set, including the 5.8S30 probe. The ordered reference oligomer of the 5.8S primer set as st*andard (10^6^ [blue], 10^5^ [orange], 10^4^ [yellow], 10^3^ [green], and 10^2^ [violet] *sequence copies) and NTC controls [grey color].*


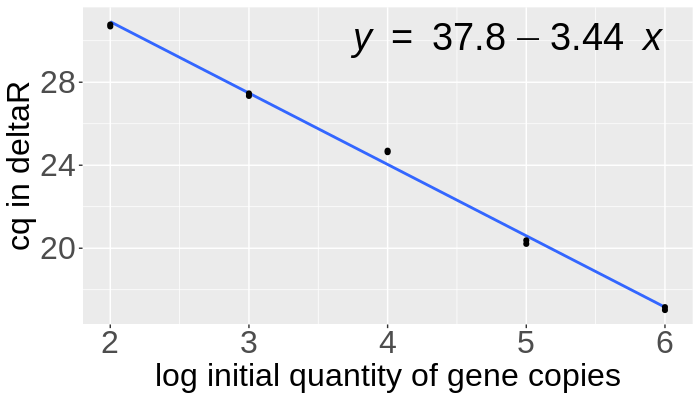

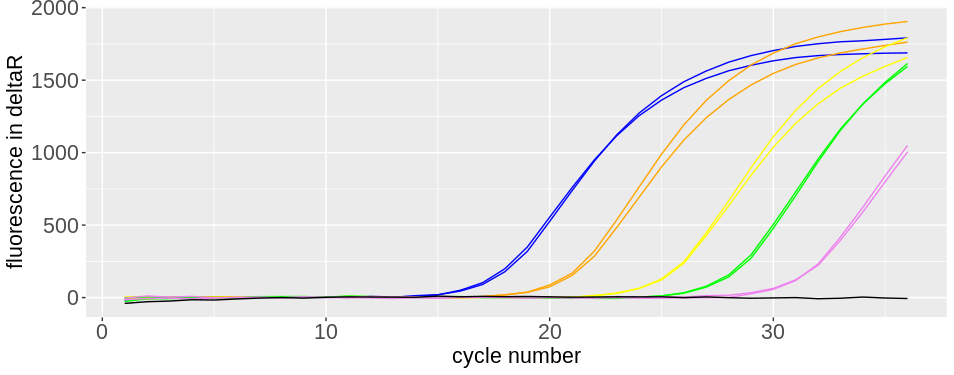

Supplement: Supplementary file 1 — Data S1. Supplementary Information. [file EMI4-16-e13257-s001.zip › Supplementals_KSP_S3_S7_S8_S9.docx]
